# Cortical Networks Underpinning Compensation of Verbal Fluency in Normal Aging

**DOI:** 10.1093/cercor/bhab052

**Published:** 2021-04-19

**Authors:** Lissett Gonzalez-Burgos, Joana B Pereira, Rosaleena Mohanty, José Barroso, Eric Westman, Daniel Ferreira

**Affiliations:** 1 Department of Clinical Psychology, Psychobiology and Methodology, Faculty of Health Science, Section of Psychology and Speech Therapy, University of La Laguna, La Laguna, Tenerife 38 200, Spain; 2 Division of Clinical Geriatrics, Center for Alzheimer Research, Department of Neurobiology, Care Sciences, and Society, Karolinska Institutet, Stockholm 141 83, Sweden; 3 Department of Neuroimaging, Centre for Neuroimaging Sciences, Institute of Psychiatry, Psychology and Neuroscience, King's College London, London WC2R 2LS, UK

**Keywords:** compensation, cortical thickness, graph theory, normal aging, phonemic fluency

## Abstract

Elucidating compensatory mechanisms underpinning phonemic fluency (PF) may help to minimize its decline due to normal aging or neurodegenerative diseases. We investigated cortical brain networks potentially underpinning compensation of age-related differences in PF. Using graph theory, we constructed networks from measures of thickness for PF, semantic, and executive–visuospatial cortical networks. A total of 267 cognitively healthy individuals were divided into younger age (YA, 38–58 years) and older age (OA, 59–79 years) groups with low performance (LP) and high performance (HP) in PF: YA-LP, YA-HP, OA-LP, OA-HP. We found that the same pattern of reduced efficiency and increased transitivity was associated with both HP (compensation) and OA (aberrant network organization) in the PF and semantic cortical networks. When compared with the OA-LP group, the higher PF performance in the OA-HP group was associated with more segregated PF and semantic cortical networks, greater participation of frontal nodes, and stronger correlations within the PF cortical network. We conclude that more segregated cortical networks with strong involvement of frontal nodes seemed to allow older adults to maintain their high PF performance. Nodal analyses and measures of strength were helpful to disentangle compensation from the aberrant network organization associated with OA.

## Introduction

Language is one of the cognitive functions that is less vulnerable to the effects of aging (Schaie and Willis 1993; Ansado et al. 2013). An explanation for this is that language abilities are broadly distributed across different brain networks (Gernsbacher and Kaschak 2003). However, some language abilities do decline with aging. For instance, verbal fluency is often described as one of the language abilities most vulnerable to the effects of aging (Kavé and Mashal 2012). Previous studies have shown that performance in phonemic fluency (PF), a modality of verbal fluency, is rather stable during the middle-age adulthood, declines around the age of 60, and tends to stabilize again after the age of 65 (Rodriguez-Aranda and Martinussen 2006; Ferreira et al. 2015; Gonzalez-Burgos et al. 2019). This age trajectory of PF may be underlain by compensatory mechanisms that are particularly functional before the age of 60.

There has been an increasing interest in compensatory mechanisms that occur during aging (Cabeza 2002; Grady 2008; Morcom and Johnson 2015; Fitzhugh et al. 2019). Compensation reflects processes through which individuals recruit brain structures, networks, or neural resources in response to brain aging, pathology, or high cognitive demand (Cabeza et al. 2018; Stern et al. 2020). Common formulations of compensation include enhancement and maintenance of performance (Cabeza et al. 2018; Stern et al. 2020). Further, individuals with higher levels of cognitive reserve (CR) may have a larger range of alternative networks or neural strategies that allow them to maintain cognitive function in a more successful manner than individuals with lower CR (Barulli and Stern 2013; Cabeza et al. 2018; Stern et al. 2020). We previously used graph theory analyses on cognitive data to investigate how CR and network efficiency contribute to PF in middle-aged and elderly individuals (Gonzalez-Burgos et al. 2020). We found that compensation in PF was facilitated by a better performance in semantic and executive–visuospatial abilities (Gonzalez-Burgos et al. 2019; Gonzalez-Burgos et al. 2020). This finding suggests the participation of ipsilateral left language networks and contralateral right frontoparietal networks in the compensation of PF, since semantic abilities have been associated with left inferior temporal, supramarginal, and frontal areas (Zhang et al. 2013; Zhang et al. 2019), and executive–visuospatial abilities have been associated with the right frontoparietal brain network, including prefrontal and supramarginal and other parietal areas (Budisavljevic et al. 2017; Nakajima et al. 2019). However, these neural correlates of compensation of PF were suggested solely from cognitive data (Gonzalez-Burgos et al. 2020), and further research is needed to elucidate whether these anatomical networks are indeed involved in compensation of PF.

The overall goal of the current study was to investigate cortical brain networks potentially underpinning compensation of age-related differences in PF. The first aim was to test whether brain regions associated with PF in previous studies (Costafreda et al. 2006; Birn et al. 2010; Tomasi and Volkow 2012; Zhang et al. 2013; Marsolais et al. 2014; Marsolais et al. 2015; Methqal et al. 2019) comprise a cortical network that is associated with performance in PF in a reference group of younger participants. Similarly, the second aim was to test whether brain regions associated with semantic and executive–visuospatial abilities in previous studies (Budisavljevic et al. 2017; Nakajima et al. 2019) comprise cortical networks that are associated with performance in semantic and executive–visuospatial tests in a reference group of younger participants. The third aim was to investigate compensation of age-related differences in PF in our older group by investigating features of these cortical networks underpinning PF, semantic, and executive–visuospatial cognitive abilities. Hence, our aim was to investigate compensation within the PF cortical network itself as well as ipsilateral compensation through the semantic cortical network and contralateral compensation through the executive–visuospatial cortical network. In this study, compensation refers to the ability to minimize the effect of age in PF in a group of older individuals and maintain high cognitive performance at levels that are comparable to those of younger middle-aged individuals. We hypothesized that older individuals with HP in PF would have a more efficient PF cortical network. Further, more efficient semantic and executive–visuospatial cortical networks would be associated with higher performance in PF in older individuals, likely delineating compensatory processes in normal aging. This study extends previous literature that has been primarily focused on declarative memory (Nyberg et al. 2012; Sala-Llonch et al. 2014; Vaqué-Alcázar et al. 2020) and functional magnetic resonance imaging (MRI) (Anthony and Lin 2018), whereas we investigated compensation in PF and used structural MRI. Unraveling compensatory mechanisms may advance our current understanding of the brain responses to both age-related and pathological processes. This may eventually help designing interventions to minimize or prevent cognitive impairment due to neurodegenerative processes.

## Materials and Methods

### Participants

A total of 267 participants were selected from the GENIC-database (Group of Neuropsychological Studies of the Canary Islands) (Machado et al. 2018), with ages between 32 and 79 years, and a balanced sex distribution across age (53% females). The sample used in the current study was selected from our previous study focused on cognitive data (*N* = 446, Gonzalez-Burgos et al. 2020), but only participants who had an MRI available were selected (*N* = 267, overlap with sample in Gonzalez-Burgos et al. 2020 is 60%). All participants were native Spanish speakers. Since language is strongly left lateralized, only right-handed participants were included in the current study.

All participants were assessed with a comprehensive neuropsychological protocol. For each participant, cognitive profile and diagnosis were established by at least 2 qualified clinical neuropsychologists, using age-, sex-, and education-adjusted normative data. Inclusion criteria for the current study were: 1) normal cognitive performance in comprehensive neuropsychological assessment (see Ferreira et al. 2015 for detailed information about the protocol) using pertinent clinical normative data (i.e., individuals with mild cognitive impairment or dementia were excluded); 2) preserved functional status and global cognition defined by a Blessed Dementia Scale (BDRS) (Blessed et al. 1968) score <4 and/or a Functional Activity Questionnaire (Pfeffer et al. 1982) score <6 and a Mini-Mental State Examination (Folstein et al. 1975) score ≥24; 3) no neurologic, psychiatric or systemic diseases; 4) no history of substance abuse; 5) no abnormal findings such as stroke, tumors, hippocampal sclerosis, etc., in MRI according to an experienced neuroradiologist. Although the BDRS scale cutoff for abnormality is frequently established at ≥4 points (Blessed et al. 1968; Erkinjuntti et al. 1988), the “changes in personality, interests, and drive” subscale may influence the BDRS total score and does not necessary reflect impairment in activities of daily living (Machado et al. 2018). With the aim of excluding only individuals with functional impairment, as an exception, we included those participants with total BDRS scores ≥4 (*n* = 16) if: 1) 70% or higher percentage of the BDRS total score resulted from changes in personality, interests, and drive subscale and 2) if a score ≤1.5 was obtained in the other 2 subscales (“changes in performance of everyday activities” and “changes in habits”). The same procedure has been used in previous studies (Machado et al. 2018; Gonzalez-Burgos et al. 2019).

The current study was approved by the Ethics Committee of the University of La Laguna (Spain), and all participants gave their written informed consent, in accordance with The Code of Ethics of the World Medical Association (Declaration of Helsinki).

### Neuropsychological Assessment

The neuropsychological protocol included tests to assess language, processing speed, attention, executive functions, verbal and visual episodic memory, procedural memory, and visuoconstructive, visuoperceptive, and visuospatial functions, as described elsewhere (Ferreira et al. 2015). Among all these tests, the PF test is of relevance to the current study. PF was assessed with the Controlled Oral Word Association Test (Benton et al. 1989). Participants had to recall words that begin with the letters F, A, and S for 1 minute each. Proper nouns, numbers, and derived words were scored as intrusion errors. A total score (F + A + S) was calculated as the number of correct words produced, excluding intrusions and perseverations (repetitions of correct words). The Boston Naming Test (BNT) (Kaplan et al. 1983), Judgment of Line Orientation Test (JLOT) (Benton et al. 1983), Visual Reproduction Test (VRT, from Wechsler Memory Scale – Third Edition) (Wechsler 1997), and Stroop Test (Golden 1978) were also included in this study. The BNT evaluates lexical access by visual confrontation. JLOT, VRT, and the Stroop Test assess visuospatial abilities, visual memory, and executive functions (inhibition), respectively. We selected these tests because a previous publication demonstrated their contribution to performance in PF, possibly underpinning compensatory processes through nonfluency networks (Gonzalez-Burgos et al. 2019). In addition, we report education level and Wechsler Adult Intelligence Scale, 3rd edition (WAIS-III) Information subtest as a measure of crystallized intelligence, for characterization of the cohort.

### MRI and Automated Image Processing

Participants were scanned using a 3 T General Electric imaging system (General Electric) located at the “Hospital Universitario de Canarias” in Tenerife, Spain. A 3-dimensional *T*_1_-weighted FSPGR (Fast Spoiled Gradient Echo) sequence was acquired in sagittal plane with the following parameters: repetition time/echo time = 8.73/1.74 ms, inversion time = 650 ms, field of view 250 × 250 mm, matrix 250 × 250 mm, flip angle 12°, slice thickness = 1 mm, voxel resolution = 1 × 1 × 1 mm. Full brain and skull coverage was required for the MRI datasets and detailed quality control was carried out on all MR images according to previously published criteria (Simmons et al. 2009). TheHiveDB Database system (Muehlboeck et al. 2014) was used to automatically preprocess the *T*_1_-weighted images with FreeSurfer 6.0.0, following standard procedures (Ferreira et al. 2014). Quality control was performed both on the original *T*_1_-weighted images (Simmons et al. 2009) and the FreeSurfer output. Original images that did not pass quality control were discarded. All FreeSurfer output passed quality control and manual edits were not needed. Among the different measures provided by FreeSurfer, we selected regional estimations of cortical thickness for the current study.

### Network Construction and Graph Analysis

**Figure 1:**
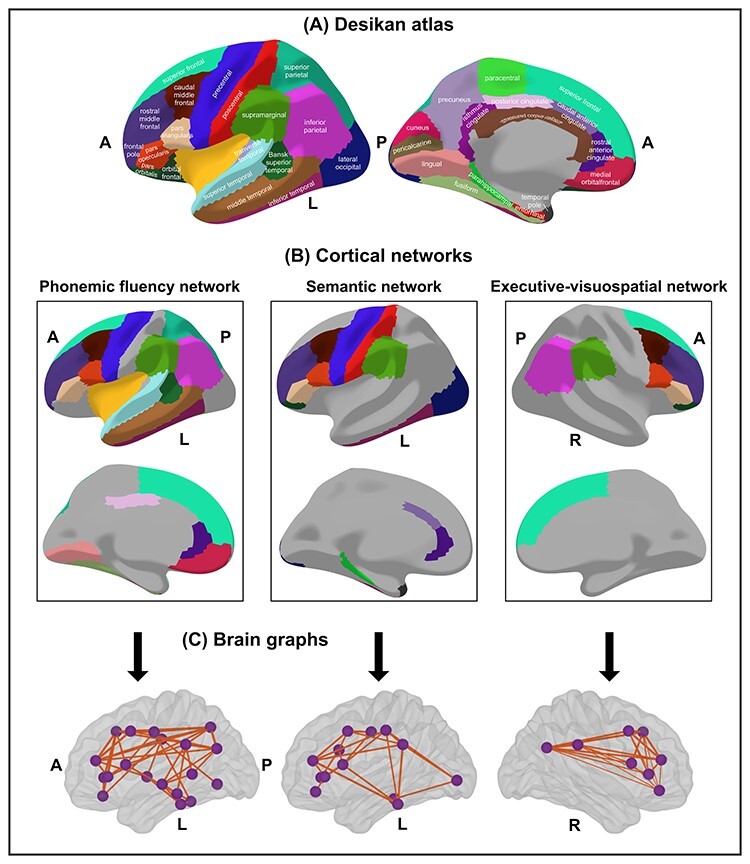
Cortical networks. (*A*) Desikan atlas; (*B*) cortical networks, cortical regions included as nodes; (*C*) brain graphs of the 3 cortical networks at the median density of 27%. Nodes are depicted as purple spheres and edges as orange lines estimated from Pearson correlation coefficients. A, anterior part of the brain; P, posterior part of the brain; L, left; R, right. The R-package ggseg3d was used for visualization (Mowinckel & Vidal-Piñeiro 2019).

The average cortical thickness from selected regions of the Desikan atlas (Desikan et al. 2006) was used as the nodes for network construction (Fig. 1). Three separate cortical networks were constructed to reflect PF, semantic, and executive–visuospatial cortical networks based on regions that have been consistently reported in previous studies (Costafreda et al. 2006; Birn et al. 2010; Tomasi and Volkow 2012; Zhang et al. 2013; Marsolais et al. 2014; Marsolais et al. 2015; Budisavljevic et al. 2017; Methqal et al. 2019; Nakajima et al. 2019). According to these previous studies, the PF cortical network mostly includes areas from the left hemisphere, although it also involves several areas from the right hemisphere. To facilitate interpretations on contralateral compensation, we limited our PF network to areas from the left hemisphere (and reserved the right executive–visuospatial network for the test on contralateral compensation). As depicted in Figure 1, the PF cortical network includes several regions of the left frontal, parietal, and temporal cortex, where the Broca (Pars triangularis), Wernicke (Bankss), and supramarginalis areas are central in well-established models of language functioning (Mesulam 1998). For completeness of information, we also ran complementary analyses for the right side of the PF cortical network (these complementary analysis as well as the nodes included in this subnetwork are shown in Supplementary Fig. S17). The semantic cortical network includes the left inferior temporal, left supramarginal, and areas of the left frontal cortex, and partially overlaps with the PF cortical network. The executive–visuospatial network includes the right prefrontal and supramarginal and other parietal areas. Although this network is expected to be highly specific to our executive–visuospatial tasks of interest, it only included 9 nodes, which may be a concern because graph analyses may be limited on small networks. Hence, we ran complementary analysis on separated larger executive and visuospatial networks (16 and 11 nodes, respectively), at the cost of specificity. These complementary analysis as well as the nodes included in these 2 larger networks are shown in Supplementary Figure S18.

**Figure 2:**
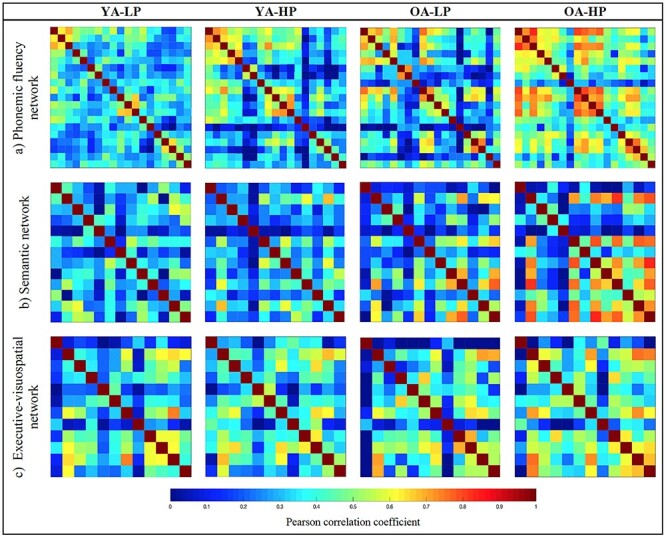
Weighted correlation matrices by study group. PF, semantic and executive–visuospatial networks.

The edges between the nodes were calculated through group-specific association matrices of Pearson correlation coefficients from each pair of nodes (Fig. 2). The matrices were binarized by thresholding the correlation coefficients at a range of densities for the 3 cortical networks (min = 10% to max = 45%, in steps of 1%), ensuring the exclusion of disconnected networks (densities below 10%) and random topologies (densities above 45%, small-world index close to 1). Network topologies were compared across this range of densities. Both self-connections and negative correlations were excluded.

Once the cortical networks were constructed, both nodal and global graph measures were calculated. Nodal measures refer to each specific node, whereas global measures refer to the average across all the nodes. Regarding global measures, we included the “average global efficiency,” “average local efficiency,” “transitivity,” and “average strength” (Rubinov and Sporns 2010). The average global efficiency is the average inverse shortest path length between a node and the rest of the network. The average global efficiency measures how efficiently information is exchanged throughout the network (Latora and Marchiori 2001). The mathematical definition of the average local efficiency is similar to that of the average global efficiency, but the average local efficiency is restricted to a given node and the subgraph created by the node’s neighbors. Conceptually, the average local efficiency is related to the clustering coefficient and transitivity measure, which can be regarded as a measure of the local efficiency of information transfer, or of the robustness of the network to deletion of individual nodes (Bullmore and Sporns 2009). The transitivity refers to the fraction of a node’s neighbors that are also neighbors of each other in the whole network, normalized by the whole network. It reflects how well the nodes are connected to nearby nodes forming cliques. The average strength is given by the sum of the weights of all edges connected to a node. The average global efficiency, average local efficiency, and transitivity measures were calculated on binary undirected networks across the different densities, and the average strength was calculated on weighted undirected networks (before binarization). In addition, the following nodal measures were calculated: the “nodal global efficiency,” the “nodal local efficiency” (Latora and Marchiori 2001), and the “nodal strength” (Barrat et al. 2004). The nodal global efficiency of a specific node is the average inverse shortest path length between that node and the rest of the network. The nodal local efficiency is the global efficiency of a node calculated on the subgraph created by the node’s neighbors. The nodal strength is given by the sum of the weights of all edges connected to a node. Modular analyses were also conducted by applying the Louvain algorithm (Blondel et al. 2008) on weighted undirected networks with a gamma value of 1. The formulae used to calculate all these graph measures are provided in Rubinov and Sporns (2010); Latora and Marchiori (2001); (Barrat et al. (2004). Network construction, measures calculation, and graph analyses were performed using BRAPH (BRain Analysis using graPH theory, www.braph.org, Mijalkov et al. 2017).

### Statistical Analysis

Statistical analyses were performed using the R programming environment (Core 2016) and BRAPH (www.braph.org, Mijalkov et al. 2017). We stratified the cohort into groups of younger and older individuals and high performance (HP) and low performance (LP) in PF, semantic (BNT), and executive–visuospatial tasks (JLOT, VRT, and Stroop, *z*-transformed and combined), using the median values of the age and selected cognitive variables. For the demographic variables, analysis of variance (ANOVA) was used for continuous and dichotomous (dummy) variables, both for main effects and for the interaction between 2 factors (i.e., the interaction between the age and performance groups). The Chi-square test was used for categorical variables. *P* values in all post hoc analyses were adjusted with the Hochberg’s (Hochberg and Benjamini 1990) method for multiple testing. Significant differences were considered when *P* ≤ 0.05 (2-tailed). Between-group comparisons of graph measures were conducted through 1000 nonparametric permutations at a range of network densities (10–45%). The 95% confidence intervals of each distribution were used as critical values for testing of the null hypothesis at *P* ≤ 0.05 (2-tailed). The false discovery rate (FDR) adjustment (Genovese et al. 2002) for multiple testing was used at *P* ≤ 0.05 (2-tailed) on the nodal measures at the median density (27%).

## Results

**Figure 3:**
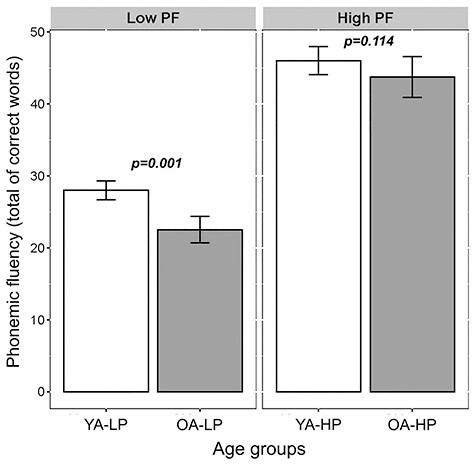
Interaction between age and performance groups with PF as the outcome measure (ANOVA). Bars represent the mean of correct words produced and the jack-knifes represent the 95% confidence intervals. Low PF, low PF performance groups; high PF, high PF performance groups. The OA group performed worse than the YA group in PF but only within the LP groups (younger adults with low PF performance, YA-LP vs. older adults with low PF performance, OA-LP, *F*_3,263_ = 136.93, *P* = 0.001).

Table 1 shows the demographic characteristics of the age and PF performance groups. The ANOVA for PF as the outcome variable did not show any significant interaction between the age and performance groups (*P* = 0.112). Subsequent analyses showed that although the older age (OA) group performed worse than the younger age (YA) group in PF; this effect was only observed within the LP groups (younger adults with low PF performance, YA-LP vs*.* older adults with low PF performance, OA-LP, *P* = 0.001) but not within the HP groups (younger adults with high PF performance, YA-HP vs*.* older adults with high PF performance, OA-HP, *P* = 0.114) (Fig. 3). Despite the group differences in education level and WAIS-III Information subtest, we did not control for the effect of these 2 variables due to our interest in compensation mechanisms, which are partly facilitated by education level and WAIS-III Information subtest (Gonzalez-Burgos et al. 2020).

**Table 1:** Demographic characteristics and cognitive performance

	**YA-LP** **(*n* = 88)**	**YA-HP** **(*n* = 85)**	**OA-LP** **(*n* = 47)**	**OA-HP** **(*n* = 47)**	***P* value**
Age	47.7 (5.5)^b^^,^^c^	48.5 (6.3)^b^^,^^c^	68.4 (5.5)^c^	65.5 (4.9)	<0.001
(Range)	(37–58)	(32–58)	(59–79)	(59–76)	
Sex (women, men)	55/33	39/46	26/21	22/25	0.12
WAIS-III Information subtest	14.4 (5.4)^a^^,^^c^	19.3 (5.4)^b^	12.5 (5.3)^c^	19.9 (4.4)	<0.001
Education level					<0.001
Illiteracy	0	0	0	0	
Unfinished primary studies	1	0	9	1	
Completed primary studies	48	16	24	13	
Completed secondary studies	23	24	9	6	
University studies	16	45	5	27	
PF	28.0 (6.2)^a^^,^^b^^,^^c^	46.0 (9.0)^b^	22.5 (6.2)^c^	43.7 (9.7)	<0.001
BNT	25.5 (3.5)^a^^,^^b^	28.0 (2.5)^b^	22.0 (5.2)^c^	26.7 (3.3)	<0.001
JLOT (first half)	13.1 (2.0)^b^	13.7 (1.6)^b^	12.1 (2.5)^c^	13.3 (1.8)	<0.001
Visual Reproduction (Immediate)	82.6 (12.3)^a^^,^^b^^,^^c^	87.5 (9.9)^b^^,^^c^	60.9 (20.1)^c^	74.4 (15.6)	<0.001
Stroop Test (Sheet 3)	37.8 (8.3)^a^^,^^b^	43.0 (9.2)^b^^,^^c^	27.2 (8.9)^c^	38.3 (8.4)	<0.001

Note: ^a^Significantly different from YA-HP.

^b^Significantly different from OA-LP.

^c^Significantly different from OA-HP.

### Cortical Brain Networks Underpinning PF and Semantic and Executive–Visuospatial Abilities—Analyses in Younger Participants

**Figure 4:**
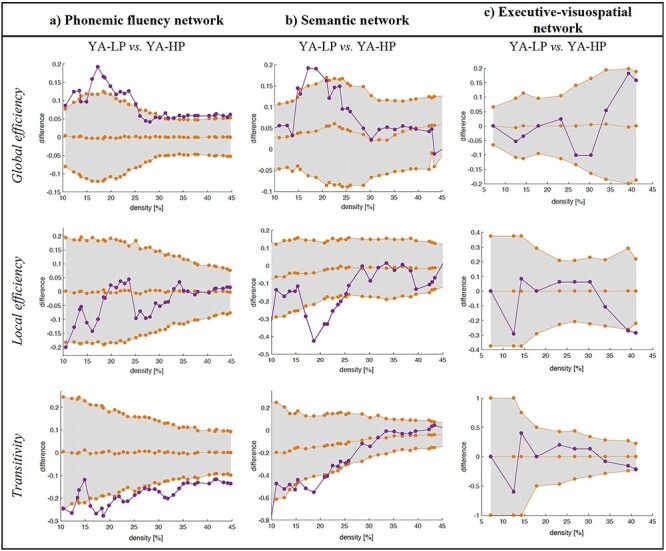
**Comparison between the young performance groups (YA-LP vs. YA-HP) across global graph measures.** a) Phonemic fluency cortical network. b) Semantic cortical network. c) Executive-visuospatial cortical network. Network densities are displayed on the *x*-axis from min  = 10% to max = 45%, in steps of 1%. Between-group differences in the global graph measures are displayed on the *y*-axis. The 95% confidence intervals were used as critical values for testing of the null hypothesis at *p* ≤ 0.05 (two-tailed), however, graphs show the one-tailed *t*-test results.

The first aim of this study was to test whether the brain regions associated to PF in previous studies (Costafreda et al. 2006; Birn et al. 2010; Tomasi and Volkow 2012; Zhang et al. 2013; Marsolais et al. 2014; Marsolais et al. 2015; Methqal et al. 2019) comprise a cortical network that is associated with performance in PF in our reference group of younger participants (32–58 years). To address this aim, we compared the YA-LP group versus YA-HP group, therefore excluding the effect of aging. For simplicity, we constrained our analyses to global graph measures. We found that the average global efficiency was increased, and the transitivity was decreased in the YA-LP group, compared with the YA-HP group (Fig. 4*A*). This finding demonstrates that this cortical network is associated with performance in PF, therefore likely underpinning PF. There were no significant group differences in the average local efficiency and the average strength. Our complementary analysis for the right nodes of the PF network showed no significant group differences when comparing the same global measures, suggesting that the PF nodes of the right hemisphere are not associated to performance in PF in our cohort (Supplementary Fig. S17).

The second aim of this study was to test whether the brain regions associated to semantic and executive–visuospatial abilities in previous studies (Budisavljevic et al. 2017; Nakajima et al. 2019) comprise cortical networks that are associated with performance in semantic and executive–visuospatial cognitive tasks, also in our reference group of younger participants. To address this aim, we compared the YA-LP group versus YA-HP group, therefore excluding the effect of aging. Regarding the semantic cortical network, the average local efficiency was decreased in the YA-LP group, and we observed a tendency for the average global efficiency to be increased and the transitivity to be decreased in the YA-LP group, compared with the YA-HP group (Fig. 4*B*). These results suggest (Fig. 4*B*) that this cortical network is likely underpinning semantic abilities. Regarding the executive–visuospatial cortical network, no differences were observed in any of the global measures when comparing the YA-LP and YA-HP groups (Fig. 4*C*). Our complementary analysis on larger separate executive and visuospatial networks showed no significant differences when comparing the YA-LP and YA-HP groups across the same global measures (Supplementary Fig. S18). This finding indicates that these cortical networks do not seem to be involved in the executive–visuospatial abilities investigated in the current study. Hence, the executive–visuospatial cortical network was not used for further analyses in this study.

### Age-Related Differences in Cortical Networks—Comparison Between Younger and Older Participants

The third aim of this study was to investigate compensation of age-related differences in PF in the older group (59–79 years) by investigating features of the PF cortical network and the semantic cortical network. To do this, we compared the older groups (OA-LP and OA-HP) versus the reference group YA-LP. In order to disentangle the effect of age from compensation effects, we tested for potential differences between the OA-HP and YA-HP groups (age) and between the OA-LP and OA-HP groups (compensation).

#### PF Cortical Network

**Figure 5:**
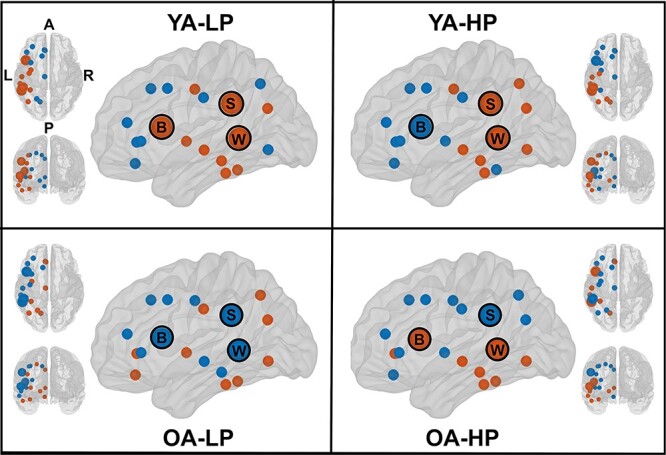
Modules—modular topology within the PF cortical network. Each module is depicted by a different color (blue vs. orange). B, Broca’s area; S, supramarginal gyrus; W, Wernicke’s area; A, anterior part of the brain; P, posterior part of the brain; L, left; R, right.

The weighted correlation matrices of the PF cortical network are displayed in Figure 2*A* (see Supplementary Figs S1–S12 for matrices with larger size and labeled regions). Visual inspection of the matrices showed that the YA-LP group had overall weak correlations (Fig. 2*A* and Supplementary Fig. S1). The YA-HP and OA-LP groups had a more segregated pattern of correlations, with a tendency for some frontal and parietal regions to correlate with each other (Fig. 2*A* and Supplementary Fig. S2–S3). The OA-HP group had the most segregated pattern of correlations, including strong correlations between several frontal and parietal regions (Fig. 2*A* and Supplementary Fig. S4). Further, we found distinct modular topologies across groups (Fig. 5). Although a total of 2 modules were identified in all 4 groups, the Broca area was included in the same module than the Wernicke and supramarginalis areas in the younger and older LP groups (YA-LP and OA-LP). In contrast, these regions were included in different modules in the younger and older HP groups (YA-HP and OA-HP).

**Figure 6:**
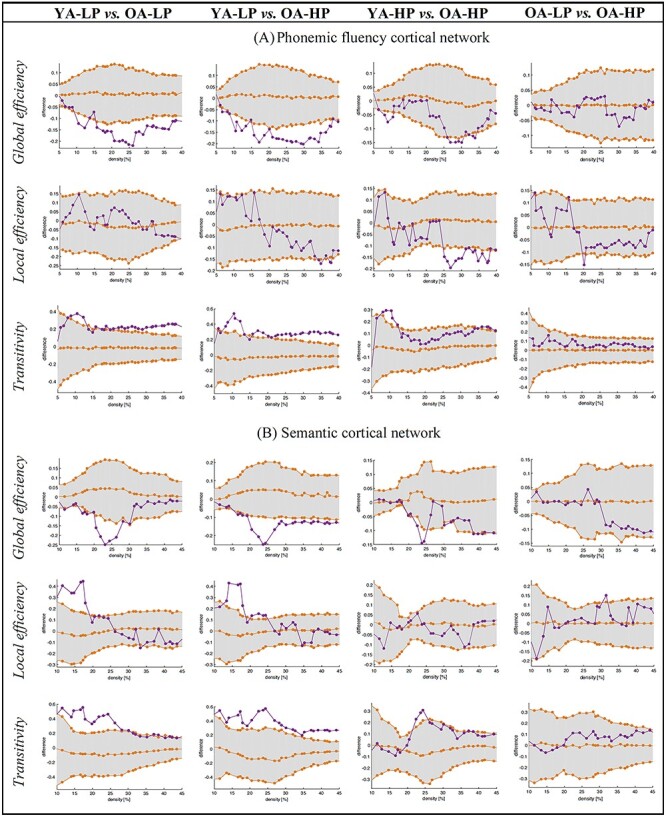
Comparison of the age and PF performance groups across global graph measures. (*A*) Comparison between PF performance and age groups across global graph measures in the PF cortical network. (*B*) Comparison between the PF performance and age groups across global graph measures in the semantic cortical network. Network densities are displayed on the *x*-axis from min = 10% to max = 45%, in steps of 1%. Between-group differences in the global graph measures are displayed on the *y*-axis. The 95% confidence intervals were used as critical values for testing of the null hypothesis at *P* ≤ 0.05 (2-tailed); however, graphs show the 1-tailed *t*-test results.

In the global network analysis, the average global efficiency was decreased and the transitivity was increased in the 2 older groups (OA-LP and OA-HP), when compared with the reference YA-LP group (Fig. 6*A*). There were no significant group differences in the average local efficiency and the average strength. To disentangle the effects of age from compensation effects, we tested for potential differences between the OA-HP and YA-HP (age) groups and between the OA-LP and OA-HP (compensation) groups. The local efficiency was decreased in the OA-HP group when compared with the YA-HP group, with a tendency toward a reduced global efficiency and an increased transitivity. The average strength was significantly higher in the OA-HP group compared with the YA-HP group (*P* = 0.035). No differences were observed when comparing the OA-LP and OA-HP groups across any of the graph measures. In the nodal network analysis, the OA-LP group showed decreased nodal global and local efficiency in the lingual and inferior temporal cortex, compared with the reference YA-LP group. The OA-HP group showed decreased nodal global and local efficiency in the lingual and cingulate cortex, and an increased nodal local efficiency and strength in the precentral and supramarginal gyri compared with the reference YA-LP group (Table 2).

**Table 2:** Nodal graph measures in the PF network

**Measure and region**		**YA-LP**	**OA-LP**	**FDR-adjusted** ***P* value**
**Global nodal efficiency**				
Left lingual		0.398	0	<0.05
Left inferior temporal		0.542	0	<0.05
**Local nodal efficiency**				
Left lingual		1	0	<0.05
**Strength**				NS
		**YA-LP**	**OA-HP**	
**Global nodal efficiency**				
Left posterior cingulate		0.630	0	<0.05
**Local nodal efficiency**				
Left lingual	1	0	<0.05	
Left rostral anterior cingulate	1	0	<0.05	
Left precentral gyrus	0	0.764	<0.05	
**Nodal strength**				
Left precentral gyrus		6.222	10.637	<0.05
Left supramarginal gyrus		6.137	11.084	<0.05

Note: NS, nonsignificant (*P* > 0.05).

In summary, the same pattern of reduced efficiency and increased transitivity was associated with both HP and OA. However, the OA-HP group reached a higher PF performance than the OA-LP group and equaled the performance of the YA-HP group (Fig. 3), likely by keeping a more segregated PF network with greater participation of frontal nodes as compared with temporal or posterior nodes in the OA-LP group and by keeping a high strength in its correlations.

#### Semantic Cortical Network

We also investigated how global connectivity features of the semantic cortical network contributed to performance in PF in the older groups, potentially illustrating compensation of PF through the semantic cortical network. To do so, global graph measures were calculated within the semantic cortical network by comparing older participants with LP and HP in PF versus the reference YA-LP group. The average global efficiency was decreased and the transitivity and average local efficiency were increased in the OA-LP and OA-HP groups when compared with the reference YA-LP group (Fig. 6*B*). No differences were observed in the average strength (see Supplementary Figs S13–S16 for the weighted correlation matrices). Again, we tested for potential differences between the OA-HP and YA-HP (age) groups and between the OA-LP and OA-HP (compensation) groups, in order to disentangle the effect of age from compensation effects. We observed a tendency toward a reduced global efficiency and an increased transitivity in the OA-HP group when compared with the YA-HP group (Fig. 6*B*). No differences were found when comparing the OA-LP and OA-HP groups across any of the graph measures.

In summary, as demonstrated for the PF cortical network, the same pattern of reduced efficiency and increased transitivity was associated with both HP and OA. However, in contrast to the OA-LP group, individuals in the OA-HP group tended to have a more segregated semantic cortical network (Figs 2*B* and 6*B*).

## Discussion

The overall aim of this study was to investigate cortical networks underpinning compensation of PF performance in normal aging. We observed a similar pattern of segregation associated with both HP and OA. Hence, 2 completely opposed levels of PF performance seem to share a common pattern of cortical connectivity. Below we discuss how these similar patterns may underlie different brain mechanisms, suggesting a successful compensation in individuals with HP and an aberrant network organization in individuals with OA and LP. Overall, older adults who performed high in PF had the most segregated PF and semantic cortical networks, involved frontal nodes more strongly, and had a high average strength in the correlations among cortical regions.

We demonstrated that isolated brain areas that have been associated with PF and semantic abilities in previous studies do comprise cortical networks underpinning PF and semantic abilities. In contrast, the right frontoparietal cortical network was not associated with performance in the executive–visuospatial cognitive tests investigated in our study. A possible explanation for this is the use of slightly different tests of executive and visuospatial abilities as well as different age groups in our study and previous studies (Budisavljevic et al. 2017; Nakajima et al. 2019). Another explanation is that the right executive–visuospatial network is a large network that involves regions of the left hemisphere as well (Budisavljevic et al. 2017; Bagarinao et al. 2019; Nakajima et al. 2019). However, we limited our network to the right hemisphere to force the distinction between ipsilateral (semantic network) and contralateral (right executive–visuospatial) compensation. Further, we restricted our right frontoparietal network to brain areas associated with the specific executive and visuospatial tasks that were previously shown to contribute to performance in PF (Gonzalez-Burgos et al. 2019, 2020). Although this restricted network is expected to be highly specific to our executive–visuospatial tasks of interest, graph analyses may be limited when conducted on small networks. Moreover, the inclusion of larger executive and visuospatial networks could increase the sensitivity and ability to find significant associations with performance in PF. However, we could not demonstrate such an association in our complementary analyses, where we tested larger right-sided executive and visuospatial networks.

Compensation can occur as a more efficient use of a specific brain network, which in our study was illustrated by network characteristics within the PF cortical network. Compensation can also occur through recruitment of other brain networks, which in our study was illustrated by network characteristics of an ipsilateral language network that is the semantic cortical network. The recruitment of a network with shared brain regions could be explained by the association between PF and other linguistic functions (Lezak et al. 2004; Gonzalez-Burgos et al. 2019). How contralateral networks such as the right executive–visuospatial network contribute to the compensation of age-related differences in PF remains to be investigated in future studies. While we limited our study to right-handed individuals as a proxy of language lateralization to the left hemisphere (Mazoyer et al. 2014), an interesting prospect is to investigate whether recruitment of contralateral networks is more prominent in individuals with a more bilateral pattern of language lateralization (Catani et al. 2007).

The main finding in this study is that the same pattern of reduced efficiency and increased transitivity in PF and semantic cortical networks was associated with both HP and OA. This finding is partially contrary to our hypothesis, which anticipated that higher performance would be associated with higher efficiency. Despite the contradictory finding on the specific measures of efficiency, it is possible that the combination of network characteristics in the HP group is indeed related to overall higher network efficiency, which was associated with higher performance. Below we discuss this interpretation further. The finding showing that groups with HP and groups with OA achieved different levels of performance in PF suggests that the implication of our study is that a common pattern of cortical connectivity may underlie different brain mechanisms. Interpreting network features in combination with level of cognitive performance is thus important. In particular, OA was associated with lower performance in PF. Hence, reduced efficiency and increased transitivity associated with lower performance in older individuals is in line with our hypothesis and suggests that this pattern of network organization is aberrant or inefficient (Logan et al. 2002; Park et al. 2004; Reuter-Lorenz and Cappell 2008; Grady 2008; Meunier et al. 2014; Cabeza et al. 2018; Vaqué-Alcázar et al. 2020). Previous studies have also reported reduced efficiency (Achard and Bullmore 2007; Sala-Llonch et al. 2014; Sala-Llonch et al. 2015) and increased transitivity (Marques et al. 2016; Farahani et al. 2019) in aging, indicating loss of specificity and effectiveness (Baltes et al. 1980; Baltes and Lindenberger 1997; Sleimen-Malkoun et al. 2014; Sleimen-Malkoun et al. 2014; Hülür et al. 2015; Pereira et al. 2018).

However, not all individuals age in the same way, highlighting between-subject variability in cognitive aging (Ferreira et al. 2017). This is clearly illustrated in our current study by a substantial number of older individuals who managed to maintain a high level of performance in PF (the OA-HP group), which was as high as the level of performance in the YA-HP group. Hence, reduced efficiency and increased transitivity associated with HP in older individuals suggests that this pattern of network organization can also be effective, possibly underlying compensatory mechanisms. This interpretation is further supported by the finding of reduced efficiency and increased transitivity associated with HP in young individuals with HP in PF (the YA-HP group). In the next paragraphs we elaborate on several findings that allowed us to further disentangle this overall network similarities related to both higher performance and OA.

Despite the overall network similarities, several findings allowed us to discriminate between compensation and aberrant network organization. We identified 2 modules in each of our 4 groups. However, the groups had distinct topological organization. The modular analyses showed that the Broca area, Wernicke area, and the supramarginal gyri were clustered in 2 separate modules in the groups with a HP. These 3 areas are central in well-established models of language functioning (Mesulam 1998). However, PF is a task primarily belonging to the so-called motor (Broca) dimension of the language function (as opposed to the sensorial —Wernicke—or transcortical—supramarginalis—dimensions). This finding in conjunction with a greater participation of frontal nodes and a higher average strength in the OA-HP group suggests that the close connectivity of the Broca area with neighboring frontal regions is preferential in order to produce HP in PF (the Broca area was segregated from Wernicke and/or supramarginalis areas in HP groups, and nodal results revealed the role of frontal nodes in the OA-HP group). Hence, this combination of features seems to illustrate a more efficient network that is associated with a higher performance, as anticipated by our hypothesis. A more segregated frontal hub with strong close connections seems to favor the higher performance in PF, which would be reflected by an increased transitivity and reduced average efficiency measures (path lengths of the frontal hub with distant brain areas may be increased due to the high segregation of the frontal hub). The findings of increased strength support this interpretation. The interaction among neighboring brain regions to reduce metabolic and wiring cost is greater in individuals with high CR (Bullmore and Sporns 2012; Marques et al. 2016; Franzmeier et al. 2018; Lee et al. 2019), who have more efficient compensatory mechanisms (Gonzalez-Burgos et al. 2020) and perform higher in PF tests (Crossley et al. 1997; Tombaugh 1999; Auriacombe et al. 2001; Roldan-Tapia et al. 2012; Gonzalez-Burgos et al. 2019; Balduino et al. 2019). The frontal lobe has been postulated as a scaffold in compensatory processes (Park and Reuter-Lorenz 2009), based on the contribution of frontal regions such as the precentral gyrus to HP in older individuals (Park and Reuter-Lorenz 2009). Despite overall age-related structural and cognitive differences reported in previous studies using the same cohort than in the current study (Ferreira et al. 2015; Ferreira et al. 2016; Machado et al. 2018; Gonzalez-Burgos et al. 2019; Cedres et al. 2019; Nemy et al. 2020), we found that a group of older participants achieved HP, comparable to that of younger adults, which presumably reflects compensatory mechanisms.

In contrast, long-distance connectivity of the Broca area seems to be less efficient and is associated with LP in PF (the Broca area was in the same community than both the Wernicke and supramarginalis areas in LP groups, and nodal results revealed the role of temporal and occipital nodes in the OA-LP group). Hence, HP in PF seems to be underpinned by a highly intraconnected subnetwork with short-distance connections, primarily including the Broca and other frontal areas. Contrarily, LP in PF seems to be characterized by the presence of a long-distance subnetwork including the Broca and other posterior brain areas such as Wernicke and supramarginal areas. Although long-distance connections can transfer information in a fast and noiseless way by reducing the path length (Buzsáki et al. 2004; Bullmore and Sporns 2012), the cost of long-distance connections can exceed its value (Achard and Bullmore 2007; Bullmore and Sporns 2012; van den Heuvel and Sporns 2013; van den Heuvel and Sporns 2019). Our findings of reduced efficiency and increased transitivity in the OA-LP group suggest that these long-distance connections are not direct in this group, which may have caused LP in PF.

This study has some limitations. We analyzed cross-sectional data; hence, our age-related differences in cognitive performance may partially be explained by cohort effects. We used a structural atlas that includes large regions (Desikan et al. 2006), and our PF network includes large regions of interest of the left hemisphere. Our current results could thus be compared with future analyses using smaller parcellations, which may perhaps provide a more fine-grained illustration of nodal contributions to compensation in PF. In addition, functional MRI and longitudinal designs may help substantiating our current results and further discriminate compensation from aberrant network organization associated to OA. Another issue is that we approached compensatory mechanisms by investigating the language function, in particular, verbal fluency. Future studies should extend our current analyses to other language components and nonlanguage cognitive functions, to inform on whether compensation is task-dependent or is a universal process (Stern et al. 2018). Finally, the software we used for graph analyses only provides the possibility to perform group comparisons. Although this is the most common form of analysis in graph studies, an approach based on correlations could introduce advantages when it comes to modeling the contribution of the semantic network to performance in PF. Future work should thus explore methods that can generate individual networks (Tijms et al. 2012), enabling correlations between graph measures and performance in PF and age as continuous variables.

Distinguishing between compensation and aberrant network organization is challenging (Shafto and Tyler 2014). Our study provides data that may help to improve this distinction. We suggest that modular analyses complemented with nodal analyses and measures of strength may help to disentangle compensation from the aberrant network organization associated with OA. Altogether, we conclude that more segregated cortical networks with a strong involvement of frontal nodes seems to allow older adults to maintain their HP in PF. Advancing our current understanding of mechanisms underlying cognitive compensation will have direct implications for the treatment and prevention of cognitive decline in normal aging and pathological processes.

## Author Contributions

Principal investigator: Prof. J.B.

L.G.B.: data curation, conceptualization, methodology writing—original draft preparation; J.P.: methodology, software, writing—original draft preparation; R.M.: methodology, writing—original draft preparation; J.B.: conceptualization, writing—reviewing and editing; E.W.: methodology, writing—reviewing and editing, supervision; D.F.: conceptualization, methodology, writing—reviewing and editing, supervision, funding. All authors contributed to manuscript revision and read and approved the submitted version.

## Notes

Data used in the preparation of this article are part of the GENIC-database (Group of Neuropsychological Studies of the Canary Islands, University of La Laguna, Spain). The following collaborators contributed to the GENIC-database but did not participate in analysis or writing of this report (in alphabetic order by family name): Nira Cedrés, Rut Correia, Patricia Díaz, Aída Figueroa, Nerea Figueroa, Eloy García, Teodoro González, Zaira González, Cathaysa Hernández, Edith Hernández, Nira Jiménez, Judith López, Cándida Lozano, Alejandra Machado, Yaiza Molina, Antonieta Nieto, María Sabucedo, Elena Sirumal, Marta Suárez, Manuel Urbano, and Pedro Velasco. *Conflict of Interest*: None declared.

## Funding

Agencia Canaria de Investigación, Innovación y Sociedad de la Información de la Consejería de Economía, Industria, Comercio y Conocimiento y por el Fondo Social Europeo (FSE) Programa Operativo Integrado de Canarias 2014-2020, Eje 3 Tema Prioritario 74 (85%); Fundación Cajacanarias; funding for Research and Geriatric Diseases at Karolinska Institutet; Demensfonden; Gamla Tjanarinnor; Gun and Bertil Stohnes.

## Supplementary Material

Supplementary_material_bhab052
