# Supplementary material for: Cortical Networks Underpinning Compensation of Verbal Fluency in Normal Aging
Source: Cereb Cortex. 2021 Apr 19;31(8):3832–45. doi: 10.1093/cercor/bhab052 (PMC8258442; doi:10.1093/cercor/bhab052)
Supplement: Supplementary_material_bhab052 [file supplementary_material_bhab052.docx]

**Supplementary Material**

**Cortical networks underpinning compensation of verbal fluency in normal aging.**

Lissett Gonzalez-Burgos^1,2^; Joana B. Pereira^2^, Rosaleena Mohanty^2^, José Barroso^1^, Eric Westman^2,3^, and Daniel Ferreira^1,2^*

**Supplementary Figure S1**. Weighted correlation matrix, phonemic fluency network (YA-LP). YA, younger age. LP, low performance. The color bar indicates the strength of the Pearson correlation coefficients: colder colors represent weaker correlations, while warmer colors represent stronger correlations.

**Supplementary Figure S2**. Weighted correlation matrix, phonemic fluency network (YA-HP). YA, younger age. HP, high performance. The color bar indicates the strength of the Pearson correlation coefficients: colder colors represent weaker correlations, while warmer colors represent stronger correlations.

**Supplementary Figure S3**. Weighted correlation matrix, phonemic fluency network (OA-LP). OA, older age. LP, low performance. The color bar indicates the strength of the Pearson correlation coefficients: colder colors represent weaker correlations, while warmer colors represent stronger correlations.

**Supplementary Figure S4**. Weighted correlation matrix, phonemic fluency network (OA-HP). OA, older age. HP, high performance. The color bar indicates the strength of the Pearson correlation coefficients: colder colors represent weaker correlations, while warmer colors represent stronger correlations.

**Supplementary Figure S5**. Weighted correlation matrix, semantic network (YA-LP). YA, younger age. LP, low performance. The color bar indicates the strength of the Pearson correlation coefficients: colder colors represent weaker correlations, while warmer colors represent stronger correlations.

**Supplementary Figure S6**. Weighted correlation matrix, semantic network (YA-HP). YA, younger age. HP, high performance. The color bar indicates the strength of the Pearson correlation coefficients: colder colors represent weaker correlations, while warmer colors represent stronger correlations.

**Supplementary Figure S7**. Weighted correlation matrix, semantic network (OA-LP). OA, older age. LP, low performance. The color bar indicates the strength of the Pearson correlation coefficients: colder colors represent weaker correlations, while warmer colors represent stronger correlations.

**Supplementary Figure S8**. Weighted correlation matrix, semantic network (OA-HP). OA, older age. HP, high performance. The color bar indicates the strength of the Pearson correlation coefficients: colder colors represent weaker correlations, while warmer colors represent stronger correlations.

**Supplementary Figure S9**. Weighted correlation matrix, fronto-parietal network (YA-LP). YA, younger age. LP, low performance. The color bar indicates the strength of the Pearson correlation coefficients: colder colors represent weaker correlations, while warmer colors represent stronger correlations.

**Supplementary Figure S10**. Weighted correlation matrix, fronto-parietal network (YA-HP). YA, younger age. HP, high performance. The color bar indicates the strength of the Pearson correlation coefficients: colder colors represent weaker correlations, while warmer colors represent stronger correlations.

**Supplementary Figure S11**. Weighted correlation matrix, fronto-parietal network (OA-LP). OA, older age. LP, low performance. The color bar indicates the strength of the Pearson correlation coefficients: colder colors represent weaker correlations, while warmer colors represent stronger correlations.

**Supplementary Figure S12**. Weighted correlation matrix, fronto-parietal network (OA-HP). OA, older age. HP, high performance. The color bar indicates the strength of the Pearson correlation coefficients: colder colors represent weaker correlations, while warmer colors represent stronger correlations.

**Supplementary Figure S13**. Weighted correlation matrix, semantic network in phonemic fluency performance groups (YA-LP). YA, younger age. LP, low performance. The color bar indicates the strength of the Pearson correlation coefficients: colder colors represent weaker correlations, while warmer colors represent stronger correlations.

**Supplementary Figure S14**. Weighted correlation matrix, semantic network in phonemic fluency performance groups (YA-HP). YA, younger age. HP, high performance. The color bar indicates the strength of the Pearson correlation coefficients: colder colors represent weaker correlations, while warmer colors represent stronger correlations.

**Supplementary Figure S15**. Weighted correlation matrix, semantic network in phonemic fluency performance groups (OA-LP). OA, older age. LP, low performance. The color bar indicates the strength of the Pearson correlation coefficients: colder colors represent weaker correlations, while warmer colors represent stronger correlations.

**Supplementary Figure S16**. Weighted correlation matrix, semantic network in phonemic fluency performance groups (OA-HP). OA, older age. HP, high performance. The color bar indicates the strength of the Pearson correlation coefficients: colder colors represent weaker correlations, while warmer colors represent stronger correlations.


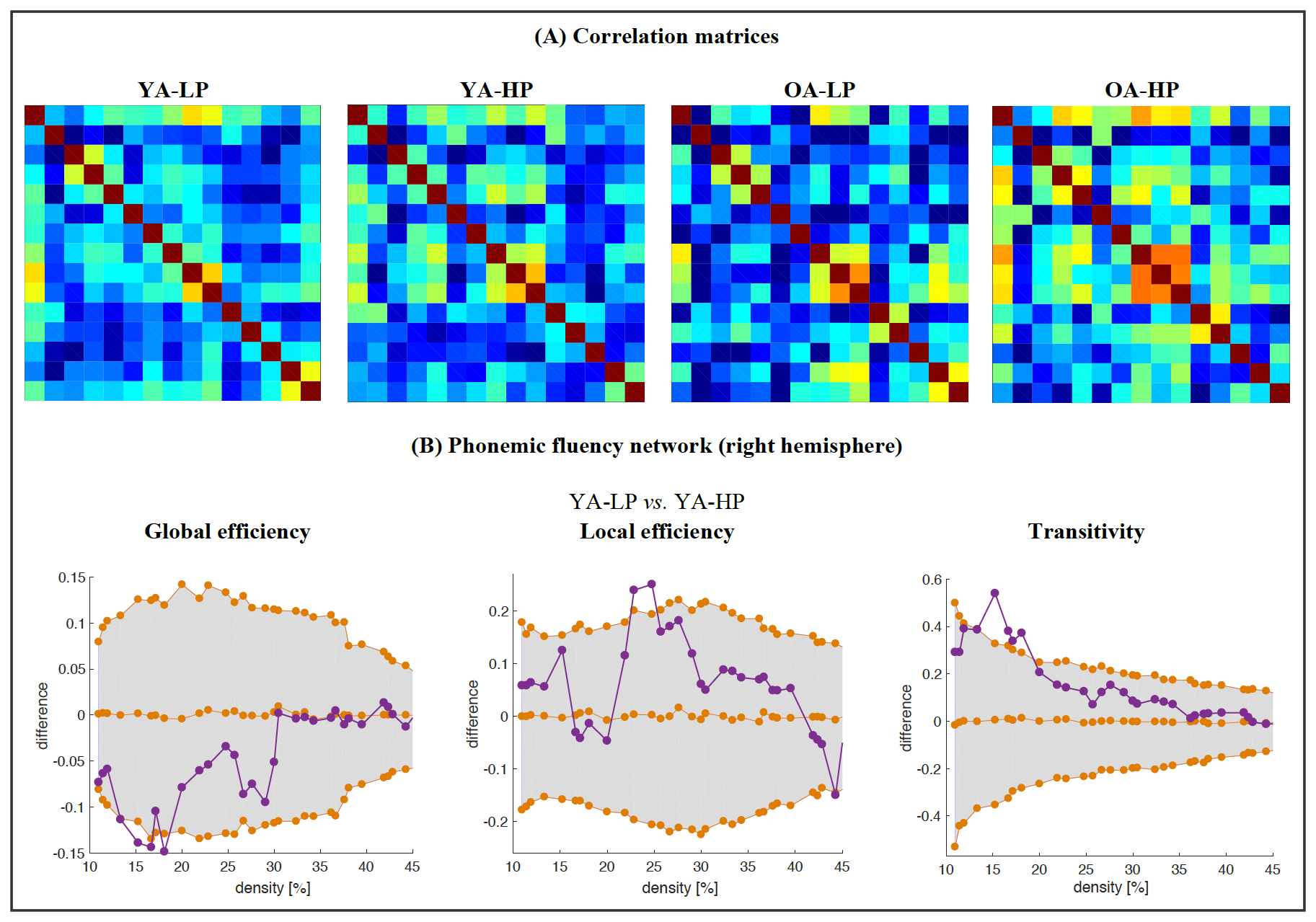


**Supplementary Figure S17**. **Phonemic fluency network (right hemisphere).** Regions included: superior frontal, pars orbitalis, pars triangularis, pars opercularis, precentral, insula, superior parietal, inferior parietal, inferior temporal, lingual, fusiform, rostral anterior cingulate, caudal anterior cingulate, posterior cingulate, isthmus cingulate, all these from the right hemisphere. (A) Weighted correlation matrices of the phonemic fluency network (right hemisphere) in phonemic fluency performance groups. YA, younger age. LP, low performance. HP, high performance. The color bar indicates the strength of the Pearson correlation coefficients: colder colors represent weaker correlations, while warmer colors represent stronger correlations. (B) Comparison between the young performance groups (YA-LP vs. YA-HP) across global graph measures. Network densities are displayed on the x-axis from min = 10% to max = 45%, in steps of 1%. Between-group differences in the global graph measures are displayed on the y-axis. The 95% confidence intervals were used as critical values for testing of the null hypothesis at *p*≤0.05 (two-tailed), however graphs show the one-tailed t-test results. There were no significant group differences in the *average strength* (YA-LP = 3.95; YA-HP = 3.80, *p*-value (two-tailed) = 0.808).


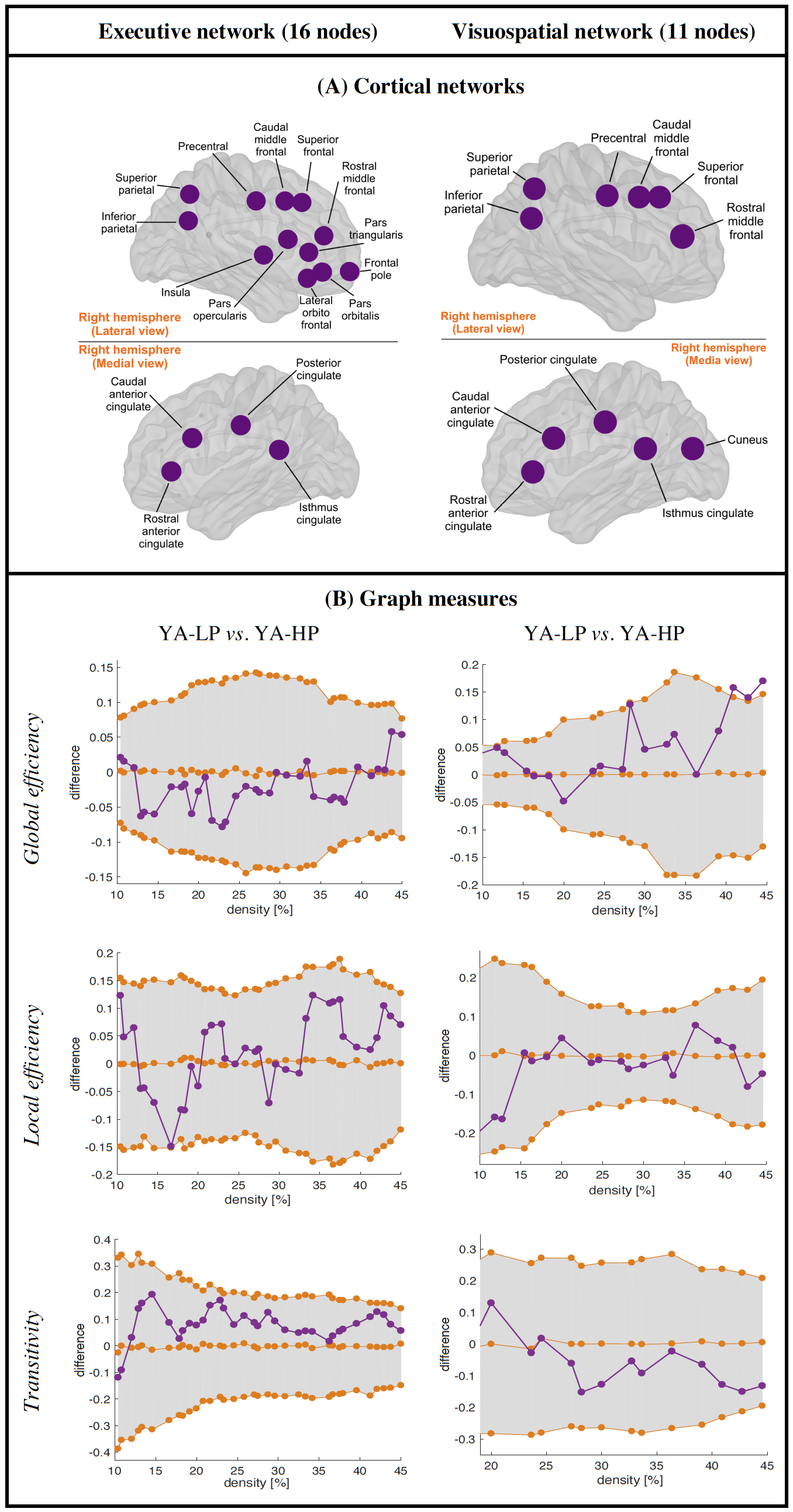


**Supplementary Figure S18**. **Comparison between the younger age – low performance and younger age - high performance groups (YA-LP vs. YA-HP) across global graph measures in the larger executive and visuospatial networks. (A)** *Cortical networks, cortical regions included as nodes. (B) Network densities are displayed on the x-axis from min = 10% to max = 45%, in steps of 1%. Between-group differences in the global graph measures are displayed on the y-axis. The 95% confidence intervals were used as critical values for testing of the null hypothesis at p≤0.05 (two-tailed), however graphs show the one-tailed t-test results.* There were no significant group differences in the *average strength* (Executive network, YA-LP = 0.502, YA-HP = 0.9960; *p* (two-tailed) = 4.523. Visuospatial network, YA-LP = 3.477, YA-HP = 3.304; *p* (two-tailed) = 0.735).
